# Clinical Surgery of the Hospital of La Pitié

**Published:** 1842-07

**Authors:** 


### BRITISH AND FOREIGN MEDICAL REVIEW,

FOR JULY, 1842.

### PART FIRST.

### Analytical and Critical Reviews.

### Art. I.

*Clinique Chirurgicale de l'Hôpital de la Pitié.* Par J. Lisfranc. Tome premier.—*Paris*, 1841. 8vo, pp. 696.

*Clinical Surgery of the Hospital of La Pitié.* By J. Lisfranc.—*Paris*, 1841.

It is generally understood that M. Lisfranc, in his own estimation, is the first surgeon of this or any other age. To the accuracy of this sweeping result, the profession at large wisely demurs; yet is not unwilling to admit that, since the death of Dupuytren, he has certainly no superior among the professors of operative surgery in the French capital, and that to his professional opinions much respect must be at all times due. He has been for many years eminent as an hospital surgeon; it seems generally admitted that the extensive field for observation afforded him in la Pitié has not been uncultivated; and when the results of such experience are laid before us by his own will and deed—not by the furtive or at all events subordinate pen of an *Interne*—they will deserve no hasty or slight perusal, and are likely soon to engross a large share of public attention. We have, accordingly, lost no time in carefully investigating the volume; and shall now endeavour to lay before our readers a practical digest of its contents, with such commentaries as the subjects naturally suggest.

Throughout many a page of this book there is entwined an amusing vein of self-sufficiency and importance; not seldom diversified, however, by snarling and sneers directed against those who venture to disagree with the author in opinion. But there is one redeeming circumstance, to which we gladly advert. Dupuytren, alive, was often loaded with reproach and abuse, as little measured as merited; dead, his name is ever associated with honour and commendation. Why should a bright spot such as this be obscured and dirtied by others of a darker and less pleasing hue? May we not hope that reform thus well begun against one blemish may be followed by removal of them all?

In the preface, after having well asserted for clinical instruction a more prominent place than is too frequently allotted to it in the curriculum of medical study,—yet admitting the necessity of its being accompanied with a sound knowledge of anatomy, physiology, pathology, and therapeutics,—he justly reprobates "concealment and exaggeration" as two most dangerous and deadly foes to the advancement of medical science; and if report speaks truly, this objurgation falls not without especial cause upon his fellow countrymen. The hint, coming from a friendly hand, will, we trust, be improved as it ought: and they will not then have to blame themselves for assisting to retard the event with an aspiration towards which he concludes his preface—"Medicine counted among the exact sciences!"—a consummation devoutly to be wished, doubtless; yet, according to present seeming, scarcely removed from the category of Utopianisms.

*On the uvula.* The first chapter of the Clinique treats of this troublesome little organ. 1. M. Lisfranc believes, with Richerand, that one function of the uvula is to forewarn the pharynx of the arrival of ingesta; by its touching this latter organ it may be supposed to create the impression on the glosso-pharyngeal nerve necessary for the production of deglutition by reflex action, as so well explained by Dr. Marshall Hall; and besides, the great number of glandular follicles which it contains may be supposed to supply mucus to favour the passage of the alimentary bolus. Thus the organ is connected with deglutition. But another duty is assigned to it by our author; viz. to prevent nasal mucosities from getting into the glottis, during emunctory efforts by strong inhalation of air through the nasal fossæ. And he states that when the uvula has been either destroyed or disabled, mucus from the nose will be found entering the air-passages, unless inspiration be effected slowly and with much caution. 2. The uvula is subject to many diseases; but the most prominent, by frequency and importance, is its elongation; when permanent, caused by paralysis of its muscular fibres. By many this affection is not treated with the respect it merits. According to the part on which the elongated uvula acts, it becomes the cause of various and important maladies; and we may not unfrequently find cases of cynanche, cough, hoarseness, nausea, and gastric irritations, which may have resisted every form of treatment, and yet, like most other affections, will at once yield to removal of their real cause. For example; an esteemed member of our profession had for several years laboured under a most distressing cough. "One day Mr. Liston happened to look down his throat, and found the uvula apparently interminable, its free end being lost in the deep fauces. A cough placed it suddenly on the dorsum of the tongue, where it uncoiled itself to the extent of fully three inches, with a fimbriated extremity, like a thin fallopian tube. By the stroke of a pair of scissors the organ was abbreviated, and the cough cut short at the same time." 3. The temporary elongations arising from excited vascular action of a chronic character, will yield readily to the nitrate of silver, or some stimulant powder, as pepper or ginger, applied directly to the part. But much mischief is done by using such stimulants indiscriminately; in acute inflammations of these parts they must materially aggravate the disease; and they ought never to be employed except when the action has been chronic from the first, or when it has been in a great measure subdued either by antiphlogistics or by time. 4. Elongation by permanent paralysis of the muscular fibres will not yield to such measures, and must be got rid of by cutting instruments. M. Lisfranc's mode of performing this little operation is nearly the same as is usually practised. He advises that it should be at once extirpated *in toto;* as, according to his experience, return of the elongation will otherwise ensue, bringing with it a necessity for repetition of the curtailment. On this point our experience differs. We have not met with such return of the disease after mere abbreviation; and even if we had, we should still encounter the risk of a second operation, rather than recklessly destroy an organ which has doubtless more than one good office to perform. 5. We cannot leave this subject without expressing our regret that the misdemeanours of the throat-cutting stammer-curers had not been made public before the penning of this memoir; for doubtless it would have been most gratifying to true surgery to have heard how soundly our author should have rated them.

*The application of leeches.* On this subject M. Lisfranc favours us with many canons: we must content ourselves with a few of them. 1. The cicatrices of leech-bites being often very apparent, we ought to refrain, if possible, from applying them to parts habitually exposed; if used there, the animals should be small. 2. In children and females of delicate skin the course of large veins should be avoided, especially in the neck. 3. Leeches on the eyelids produce unseemly ecchymosis, and often an œdematous erysipelas; they should be placed, instead, on the temple, along the roots of the hair, or behind the ears. This statement we think is too sweeping. To the general eyelid we grant that leeches are inapplicable, for the reasons stated; but we are in the habit of placing them on the inner angle, immediately beneath the tendon of the orbicularis—limiting them to that spot; and not only do we get much blood, but besides untoward results are a rarity. 4. Leeches to the inner surface of the eyelids are ineffectual as evacuants, and the bites prove injuriously irritant. Consequently, scarifications are here preferable. 5. In inflammation of the fauces, leeches should be placed over the mastoid processes or behind them; there the results are not seen, and moderate pressure readily commands bleeding. 6. In applying leeches to the epigastrium, let none fasten over the costal cartilages; otherwise, the movement of these is likely to entail a troublesome bleeding. M. Lisfranc has known it prove fatal. 7. In leeching a part where there is much subcutaneous fat but little blood will flow; in such circumstances therefore it will be prudent to increase the number of leeches or aid them by venesection. 8. Do not place leeches where there are many subcutaneous nerves; the pain will be great; erysipelas may result. For example, in leeching the forearm, prefer the dorsal to the palmar aspect. 9. Leeches should not be applied to the mucous membrane of the vulva, nor to the immediate neighbourhood of the rectum; the bites are apt to degenerate into troublesome ulcers; applied round the margin they are equally potent as remedial agents. 10. The scrotum, prepuce, the skin of the penis, should not be directly leeched; the pain is excessive; inflammation and gangrene have resulted; when the leeches are placed behind the scrotum on the raphe, the result is in every way satisfactory. 13. By leeching the skin investing the mamma great pain is occasioned, and erysipelas not unfrequently results; the surrounding integument is the preferable site.

14. If possible, leeching of inflamed skin ought to be avoided. Here we most cordially agree with M. Lisfranc, and fear not to pronounce that leeching in erysipelas, for example, is far too frequent in practice; as in the eyelids, it fails as an evacuant, and proves a direct irritant; thereby aggravating the evil. When local abstraction of blood is demanded, in such circumstances, leeches are to be superseded by punctures and incisions, according as the nature of the individual case may require. 16. Leech-bites on a syphilitic bubo are, according to the experience of M. Lisfranc, liable to ulcerate and assume the venereal characters; the occurrence, however, he admits to be rare. 17. Do not leech a fractured limb at the site of the injury, otherwise degeneration of the bites may materially interfere with the efficiency of the retentive apparatus. 18. Leeching seems to be a favorite mode whereby M. Lisfranc combats strangulation of hernia; for obvious reasons, the leeches are not applied over the tumour, but in its neighbourhood. 19. When in doubt as to the nature of a tumour, leeches may sometimes seem a good tentative application. Do not apply them *to* the tumour, however, but *near* it; otherwise, should the swelling prove carcinomatous, these leech-bites may be the means of accelerating the open or advanced condition of that loathsome disease.

Touching this subject of leeching tumours, simple or not, with the view of obtaining absorption of the adventitious growth—we are sorry to find M. Lisfranc apparently so pleased with the proceeding. We hate it altogether, along with the whole catalogue of absorbents and discutients in such circumstances: having fully made up our minds not only as to their total inefficiency to do what is expected of them, but also as to the certainty of their doing evil; and we gladly avail ourselves of this opportunity to record our opinion upon the subject. It is only on the simplest form of tumour, the mere enlargement of texture, or simple sarcoma, that leeching and discutients can have any beneficial effect. This is the only adventitious growth that will or can yield to discutients. Some others may have their onward progress delayed by occasional leechings; but this is fruitless as to cure; and, as consuming valuable time which ought to have been employed in the only radical mode of treatment, the practice becomes absolutely hurtful. All other tumours are injured by counter-irritants, and other so-called discutients of the active class; for instead of stimulating the absorbents alone, they also, and more especially, excite the perverted *nutritive* action to further activity; the tumour grows apace; forms new attachments; stretches through its capsule; becomes incorporated with new parts; and, what is worse, is certain sooner or later to degenerate as to the character of perverted deposit—from sarcomatous to carcinomatous, encephaloid, melanoid or fungus hæmatodes, or these evil products in varied combination. By suppuration, a scrofulous tumour is broken down; and, after the integuments have given way, it may gradually disappear by disintegration. A fibrous or a fatty tumour may be isolated from its connexions by a suppurative inflammation, and come away in a sphacelus. But such fortunate accidents are rare even to such tumours; and none of these will discuss by absorption. How often do we see this broad and important principle for which we contend illustrated in the theatres of our hospitals? A patient is placed there for excision of a tumour, the integuments over which are seamed and scarred by leech-bites, pustules of tartar emetic, issues, cauterizations, flayings by acid, &c.; whose history at its origin was one of an obviously simple and then innocuous formation. Then it could have been removed almost by simple incision ; but now it will require a tedious, difficult, and dangerous dissection ; and its section after all will probably display a structure of such an unpromising aspect as to give a very gloomy character to the prognosis. All this untoward chain of events is in many cases solely the result of discutient treatment, grossly misapplied ; and the sooner such untoward attempts are extruded from the practice of surgery the better it will be for suffering humanity.*

*On bloodletting in general.* We extract the more prominent of M. Lisfranc's remarks. 1. General bleeding is especially adapted to inflammation of the parenchymatous organs, local bleeding to those of the membranous tissues; the one being mainly under the influence of capillary circulation, the other under that of the larger vessels. 2. A healthy system will not bear bleeding so well as the inflammatory; a tolerance of the remedy is induced by acute inflammation ;—but only by the acute form, for after its declension bleeding is not well borne, and therefore ought not to be recklessly practised at that period. 5. Inflammation is sometimes so acute as seemingly to concentrate all vital power on the organ affected ; thus the patient may be placed in a state of extreme feebleness and depression ; and yet bleeding is rigorously demanded to save life and organic change. 6. Females repair loss of blood with extraordinary facility; they consequently require, comparatively, more frequent bleedings, and greater attention to subsequent regimen. 7. When acute inflammation has supervened on chronic, and when by bleeding the pulse and patient are already enfeebled,—as a general rule, bleed no more, although the disease be unconquered. 8. In old people, when organs previously sound are attacked by acute inflammation, and when the symptoms are of a sthenic character, cautious bleeding is advisable. But when the action has been preceded by one of a chronic nature, bleeding is dangerous. 9. In traumatic inflammations, bloodletting is especially useful; and by attending to this principle more than his followers, M. Lisfranc conceives that the result of injuries treated in La Pitié is remarkably successful. In penetrating wounds of the chest, particularly, severe bleeding is supremely advantageous, as proved by the experience of military surgeons. 13. In inflammation of the brain or its membranes, bleeding requires caution, otherwise " the equilibrium which ought to exist between the nervous and sanguineous systems may be destroyed, by excitement of the latter." 14. Abdominal inflammations soon induce prostration of the system inimical to full bleeding. This prostration is the result of a poisoning effect on the system, occasioned by absorption of gaseous and liquid putrescences generated in the deranged digestive organs. 17. Bleeding is not only useful as evacuant and antiphlogistic in large doses ; but is also, in smaller, revulsive. Three ounces will not enfeeble the system, and yet do much good to a local disease, by a derivative effect. A few leeches placed at a distance from the affected part draw blood *from* it *to* the

* An apparent exception to this general rule is explained when treating of the extirpation of carcinoma.

site of the abstraction; and according to the place of application, *a few* of these animals may thus be made either stimulant or sedative to a local and depraved circulation. 18. On the foregoing principles, chronic affections of the uterus have been successfully treated by M. Lisfranc, by small revulsive bleedings from the arm; these are supposed to produce a congestion in the upper parts of the body, thereby relieving the lower; and accordingly while the uterus is relieved, headach, dyspnœa, and palpitation are found to occur, annoying the patient considerably for a short period. In some few cases, however, this mode of treatment failed to bring relief.

*On the stethoscope as a means of diagnosis in fracture, &c.* Shortly after the discovery of auscultation, M. Lisfranc employed the stethoscope in surgical diagnosis, and published a small work on the subject. He used this instrument to detect fractures on the dead subject; and being abundantly satisfied with the result, has now, after long experience, proclaimed it as perhaps the most useful means in the diagnosis of this class of injury. In fact he seems prepared to maintain that there need be henceforth no doubtful case, excepting certain breaks of the cranium, and mere fissures of bone. With the stethoscope, swelling is never so great as to conceal crepitation; while for its detection—and this is fully as important—very light movements suffice. The surgeon is saved from the horrors of error in diagnosis; the patient is spared much torture of manipulation, grievous in itself, and disastrous in its results. 1. Close to the fracture, it is immaterial whether the instrument be used entire or not; but when applied at a distance from the fractured point, it ought to be without its stopper. 2. The more superficial the bone, the more distinct the crepitation, and the slighter the movement required for its detection. 3. When there is *riding* of the fractured ends, crepitation is of course not so easily recognized: moderate extension and counter-extension will render it distinct. 4. Crepitation produced by the fragments of a compact bone are very sharp and strong, and sometimes positively painful to the ear. 5. Crepitation from fragments of a spongy bone is dull, and resembles the action of a file on a hard yet porous substance. 6. In oblique fractures, it is stronger than in transverse. 7. When fluids are effused around the fracture, besides crepitation we have a noise resembling that which the foot produces in a leaky shoe during wet weather. 8. When the fracture is complicated with splintering, crepitation is accompanied with a noise resembling that made by angular hard bodies rubbing against each other. 9. When fracture is combined with wound of the soft parts, besides crepitation we have sounds resembling those made during strong expiration and inspiration, the mouth remaining wide open. 10. Dislocation cannot be confounded with fracture; for the noise produced by displaced articulating surfaces is light, dull, limited, and plainly caused by polished and moist surfaces moving on each other. The working of tendons in their sheaths is still less likely to be mistaken. 11. As the eye with the microscope, so the ear with the stethoscope, is not to be trusted until after a preliminary *education.* 12. In regard to fractures of the radius, Lisfranc agrees with the majority of experimental surgeons in thinking that Dupuytren has been too decided in his declaration as to the impossibility of luxation of the wrist occurring without fracture of the distal extremity of the radius. That such a combination usually exists, is beyond doubt; yet it seems equally well ascertained that such combination is not invariably essential to the existence of the dislocation. Cases in illustration are stated by M. Lisfranc, in support of this view; with most surgeons, as already stated, it scarcely needed this corroboration. 13. He also lends the weight of his authority to the occurrence of flexion of the long bones, especially of those in the forearm, in children, and even in adolescents as far advanced as fifteen or eighteen years, without fracture; and that such bending will prove permanent if not suitably treated, muscular action alone being totally inadequate to remove the deformity. Lately, traces of skirmishing on this subject were to be found scattered in the journals of this country. But we have not considered it necessary, now or then, to grapple seriously with the question; being convinced that every experienced and judicious surgeon has long since embraced the opinion as here stated. 14. Detection of stone in the bladder by sounding, even in these days, is not exempt from fallacy; and M. Lisfranc with his stethoscope is likely to aid in the investigation. Desault mistook a fungous tumour of the bladder for a stone; Dupuytren cut into the bladder, and found no foreign body there but the point of his own finger; and similar blank drawings of the covert have occurred in our own experience. According to our author, the stethoscope is to be placed on either the pubes or the sacrum; if the sound be then introduced and made to impinge on a stone, the *click* will be heard so very distinctly, under any circumstances, as to render the occurrence of mistake in diagnosis almost impossible. 15. Biliary calculi are *sometimes* to be detected by the stethoscope; our author in one case by its careful use was sensible of a noise, in the site of the gall-bladder, resembling that produced by small stones firmly impacted and rubbing on each other; and inspection after death showed three small gall-stones in the usual site. 16. M. Lisfranc would also extend the use of the stethoscope to sonorous foreign substances, lodged, or suspected to be lodged, in various parts of the body. For foreign matter in the air-passages, it has long been in efficient use.

*Fractures and their treatment.* 1. Some people, as we have already stated, took the liberty of asserting that bending without fracture did not or could not occur. We have already rebutted them; and now do the same good office to those who may venture to deny that a bone is sometimes partially fractured; that is, bent to a considerable extent, with more or less solution of its continuity in the osseous fibres at the convexity of the curve. Such accidents are not unfrequent; their peculiarity is that when by suitable manipulation the curve has been effaced or nearly so, crepitation then becomes distinct. Mr. Liston in his work on Surgery dwells particularly on this form of accident. M. Lisfranc entertains similar opinions on the subject. Sceptics have taken another step, as if desirous of securing unity in fracture, as in the arts and the drama, and have denied the existence of longitudinal fracture; apparently insisting that all bones shall break in one uniform manner, consistent with their own peculiar creed. With M. Lisfranc we agree in disagreeing with them, appealing to the evidences of museums, especially those enriched from military practice.

2. In regard to the use of retentive apparatus in treating fractures, there are two extremes of practice—delay, and severity of early application. M. Lisfranc, we think, inclines too much to the former. We are ready to admit that nothing can be more injudicious than the tight rolling of a bandage on a much shattered limb soon after infliction of the injury, especially if the apparatus be placed with the intention that it shall remain for some considerable time unlooked to and undisturbed, and more especially if the degree of pressure be not uniform, as is too frequently the case; inflammation, abscess, gangrene, are most likely to be thus induced, of course with severe attendant injury to the system; and even failing these, experiment has shown that tight pressure over the site of fracture is likely to prevent the formation of callus. Nevertheless we are equally prepared to contend that there is no more effectual means of kindling and keeping up inordinate excited action in the part, than jactitation of the broken fragments by the voluntary and involuntary movements of the unrestrained limb, and the consequent increase of injury which such motion cannot fail to occasion; inflammation, abscess, gangrene are just as likely to result from this extreme as from the opposite; if abscess form, and suppuration continue, callus will not form, or will at least be deficient—for inflammation proper is as hostile to the formation of callus, as to the healing of a flesh wound either by adhesion or granulation; if the accident stop short of this, the callus will probably prove greatly excessive, and more or less deformity will inevitably result. And therefore we agree neither with M. Lisfranc nor with his antagonists on this point; but along with, as we believe, the majority of good surgeons, place the limb immediately in apposition, and retain it so, by suitable retentive apparatus—applied loosely, so as merely to restrain motion; and often looked to, and shifted if necessary, in order that no undue pressure may be exercised upon the limb, and especially on any isolated portion of it. This we maintain to be common sense, and we believe it to be common surgery now-a-days, at least upon this side of the channel. In brief, we are advocates for immediate application of retentive apparatus, light, suitable, and temporary; and of such a nature as to admit of modifications of tightness being readily accomplished, without painful and injurious jarring of the fracture. Of course, the more severe the injury, the more guarded and watchful the use of compression in any form, and with any view.

3. In prophylactic treatment addressed to the general system, M. Lisfranc is very energetic. He bleeds from the arm frequently, during the early period of treatment, and, he says, with the best effects, in those cases of severe injury where undue vascular excitement is probable. We agree with him thoroughly in principle, but may differ slightly as to the mode of obtaining the result; and are accordingly inclined to doubt that an imperative necessity exists for so copious an abstraction of the vital fluid as he would indicate. With one well-timed bleeding, followed by antimonials, sedatives, &c., and with bread and water as the staple commodity of diet, we believe that inflammation and its deleterious consequences may be warded off in a case of bad fracture just as effectually as by the more heroic treatment of M. Lisfranc, and with far less injury to the constitution. One principle in his bleedings is a good one; to choose the site of venesection as far as possible from that of the inflammation; that the remedy may not only be depletory but revulsive. And whether we agree with him or not as to the extent of such depletion, we are free to admit that he has done good service to surgery by turning attention more closely to the subject; being fully convinced that by many otherwise excellent surgeons *such* treatment is too much neglected, to the detriment of both the patient's safety and their own credit; confining their attention almost solely to the local treatment, thereby doing but half their work; and seldom thinking of the general system at all, until it is too late to avert the mischief which uncared for disturbance there has induced. We often find that people far advanced in life make remarkable recoveries from fractures tolerably severe. It is by nature doing for them what the surgeon ought to do for adults; warding off inflammatory action—the great foe of union both in the soft and hard tissues.

4. If it be wished to abstract blood from the site of the injury, do not apply leeches directly to the part; otherwise the bites are sure to prove troublesome.

5. When fracture is complicated with wound, cease from bleeding and other evacuant treatment, when pus is about to form; otherwise, according to M. Lisfranc, you will induce reabsorption of the pus, and sad effects upon the system. However explained, this is a sound practical precept. More especially, let evacuant treatment cease when pus is profusely secreted; for now the patient is fast approaching, if he have not already attained, a time when all the resources of his system will be called upon to bring him through his trials and difficulties; and when further reduction of these resources by injudicious medico-chirurgery, would probably entail loss of the limb, of life, perchance of both.

6. As to position; a broken limb should generally be placed so as to relax the muscles implicated, for obvious reasons; hence the posture of semiflexion will be found the most generally applicable. Doubtless; but only when this is compatible with other indications of treatment, especially the maintaining of the due length of the limb. Accordingly, we disagree with M. Lisfranc in treating fractures high in the thigh with the limb semiflexed, being convinced by experience that it is only by permanent extension and counterextension as obtained by Desault's splint in the straight position, that shortening of the limb, and that to a considerable extent, can be prevented. At the same time we agree with him as to the imperious necessity there exists for great watchfulness in the use of this apparatus, in old and delicately framed patients; taking care that the pressure may be at no time excessive, otherwise sloughing and even fatal results may ensue. There is nothing good in this world whose use may not be recklessly and readily converted into abuse; so is it with many good rules of surgery; and this is one. Place then your limb in a relaxed and comfortable posture if possible; but rather let the attitude be a fatiguing one than encounter the risk of a permanent deformity with impairment of function.

7. Sometimes when, *à priori,* we imagine the half-bent position will prove eminently useful, we are deceived. Thus, while fracture in the upper third of the leg obviously demands the straight posture, fracture in the lower third is most especially suited to that of semiflexion; yet cases will now and then occur, in the latter situation, as we have ourselves experienced, when, on account of some peculiarity of the fracture, apposition cannot be satisfactorily obtained until the limb is placed straight, and kept so. Failing the one posture then, we must try the other; and not walk blindly in the beaten track, either here or elsewhere.

8. It is the custom of some surgeons to moisten their retentive apparatus, and some use stimulating fluids for this purpose. Nothing can be worse; we do not wish to stimulate the part, but to soothe it. And therefore if the moistening system is to be pursued, let the fluids be as bland as possible.

9. During the first and second weeks, M. Lisfranc *looks at* his fractures every twenty-four hours, or at furthest once in the two days; and he is right. But we are afraid of a false reading here; substituting movement and rough manipulation, for mere watchfulness of the tenderest kind. All meddlesome surgery is bad; and in no class of cases is it more injurious than in fractures. A broken bone being given, and the means asked whereby union may be most effectually prevented, we answer—daily movement of the fractured portions. We knew a surgeon who treated a fracture with great attention, and at the expiry of the usual period of probation granted to such cases, he complained lustily that there was still no union even begun, although he had every day taken care to ascertain whether the process had commenced or not, by imparting free motion to the broken surfaces. To his great sorrow, and the patient's sad detriment, motion between these continued to be as easy and free as on the first day of the injury. It would indeed have been very surprising had the result proved in any way different. We cannot satisfy ourselves too often that all is advancing favorably at the site of fracture, but, at the same time, we cannot too seldom interfere with the position of the limb. There is a wide difference between examining a part, and subjecting it to rude, unnecessary, and injurious manipulation.

10. In compound fractures, a small quantity of pus, cooped up in the interior, may induce the gravest results. This is a most important and sound maxim, and ought to be much more generally acted on than it is. As soon as the matter has formed, the sooner its evacuation is accomplished the better. To talk of delay, and digestion, and maturation, in such circumstances, savours of puerility or senility, or both.

11. Splinters when small, and by adherence to soft parts likely to retain vitality, are to be replaced not removed. M. Lisfranc adduces a case in point; there are many such.

12. M. Lisfranc says that he has seen splinters absorbed. If they remained alive, he is partly right; they may have been vitally disintegrated. But if they died, and became sequestra, we hold him to be unwittingly guilty of an untruth; they could not then be got rid of but by extrusion. As well might he hope to obtain absorption of a bullet or stone. If a settlement of that question ever were required, it was obtained from the recorded experiments of Mr. Gulliver.

13. Remember that the callus often remains comparatively soft and pliable for a considerable period, and that therefore deformities by maladjustment are not to be despaired of, at the end of six weeks or more; suitable means to improve the apposition are instantly to be adopted, and patiently persevered in: not unfrequently, unlooked for success will reward the labour.

14. As diuretics are used for dropsies, so does M. Lisfranc employ them to remove obstinate accumulations of blood, resulting from fracture.

We are not quite sure about his reasoning on this point, but he says that in his practice, such treatment expedites absorption of the effusion. He exhibits purgatives with the same view. But, as a general rule, purgatives are inapplicable to fracture; they move not the bowels alone, but the limb also; while they may do good, they must do harm; and consequently are to be avoided.

15. For ununited fracture, the preferable treatment is continuance, doubly vigilant and accurate, of retentive measures; the only difference between the treatment of this and ordinary fracture being—that while the one requires but weeks, the other takes months for completion of the cure.

16. There are fashions in surgery as in other matters; and, at present, the starched bandage is all the rage in fractures. This caprice is rebuked by M. Lisfranc, and we think most justly. We do not object to this form of bandaging *per se*, but to its indiscriminate use. During the first period of severe fracture, it is inapplicable; for considerable swelling must occur, requiring proportionate slackening of retentive apparatus, which ought consequently to be light and easily changed. Further on in the case, when the swelling has reached its acme and begun to subside, the starched bandage is still inappropriate; if applied to-day, the limb will have shrunk so far by to-morrow that the apparatus has ceased to be retentive, and the ends of the bones may ride each other at pleasure. If employed at all then, it must be almost daily renewed, and that is foreign to the nature of the application. It is only during the later periods that its use becomes judicious. When the time for inflammatory swelling has gone by, and when further atrophy of the more or less swollen limb is improbable—then the permanent, fixed, and unyielding nature of the application ceases to be detrimental, and becomes most salutary. If it be used sooner, it ought not to be in mass, but after bisection, so that the apparatus then comes to resemble two neatly and closely fitting splints of the ordinary kind. But, on the whole, we believe that such splints will be found more advantageous in diseases of the joints—chronic and acute, curable and hopeless—by obtaining the all important *rest* of the diseased parts. It is by better attention to this point by such means, that superior success has been attained by modern surgery in this sphere of disease.

17. In fracture of the ribs, M. Lisfranc is not contented with mere bandaging of the chest, but by placing compresses over the sternum, takes care that the bandage shall exert its pressure chiefly in the antero-posterior axis, throwing out the arch of the ribs, and thus keeping the broken fragments not only more fixed, but in a more accurate position. He also most properly insists on the pad for fracture of the neck of the humerus being placed well within the axilla, otherwise it must do more harm than good.

18. Fractures of the forearm are to be treated not only by the usual long splints, but also by compresses placed in the interosseous space, to prevent undue approximation of the broken ends in that direction. The same principle extends to fractures of the metacarpal bones. In the latter case, the arched form of the part must also be kept in remembrance.

19. Dupuytren's splint for broken fibula is acknowledged faultless, even by Lisfranc.

*Some cancers, apt to be thought deep, are superficial.* Some textures seem to resist cancerous disease more stoutly than others; for example, fibrous membranes, not excluding the peritoneum and pleura. By observing this, our author was led to suspect certain affections, either malignant or seeming to be so, of being more limited than is generally imagined; by acting on this idea, he states that he has been able to save important textures which would otherwise have been sacrificed by the knife; and that, after such limited operations, the disease has not returned. 1. In apparent cancer of the penis, for example, by a preliminary incision having ascertained that the sheath of the organ is yet intact, he has simply dissected off, very carefully of course, the superficial disease, leaving the important parts beneath little if at all impaired in their functions. With this result he is the more especially delighted, having found in all his experience that amputation of the penis, whether in young or old, is invariably followed by dulness, inactivity, despondency; in fact, by complete emasculation in every sense of the term. Further, he says most truly, "the true end of surgery is to preserve, not to destroy." 2. In apparent cancer of the tongue he has been able, on the same principle, to save that truly important organ, (his first case is that of an advocate, a class to whom that dangerous little member is peculiarly valuable), by dissecting off disease not involving the muscular texture, instead of, after the usual fashion, removing the whole or a large portion of the tongue, by knife or ligature. 3. In the vagina and rectum, in the eyelids and eyebrows, over the loins, and in other situations, he has followed similar practice with success. 4. In many cases he states that cancerous affections of the nose do not extend so deeply as to involve the cartilages, and that consequently ablation of their coverings ought to supersede amputation of the whole organ. The cartilaginous texture, he says, granulates kindly enough.

Now, the foregoing is an excellent principle to have mooted, but the practice requires confirmation by others than M. Lisfranc. He is well known to be too easily persuaded of the cancerous nature of morbid products, and therefore we have guardedly termed the affections to which he here alludes, as "apparently cancerous." For the cases are far too loosely described, and the description which is given far too unsatisfactory, for almost any one but the author to admit that the disease is even malignant. If it were so, only in its incipient stage could such limited dissection prevail against it. And, most certainly, we are not to be induced, by any such reasoning, to forego the ancient and sound maxim of surgery, to cut as widely of actual carcinoma, and more especially of cancer, as circumstances will allow. When you cut (for cancer) do not cut close.

*Some remarks on cancer.* 1. M. Lisfranc is satisfied, by actual experiment, that the disease is not contagious. 2. But, scoffing too much at the idea of general infection of the system by the disease, he is led to espouse sanguinary practice in advanced cases of cancer, inconsistent with prudence and humanity. 3. Cancer apparently the result of external causes is less liable to return after operation than when the disease has seemed idiopathic. 4. Permanent pimples of the face are harmless in youth, and may remain so in old age, if not irritated; if injured, they are apt to degenerate, and to assume the characters of malignant action: as soon as such degeneration threatens, they are to be extirpated. Would it not be wise to go a step further, and root them out at a still earlier period? It is always better to prevent than to cure. 5. The bistoury is preferable to caustic as the agent of removal. The latter is more painful and less certain; sometimes failing to destroy the whole morbid structure, sometimes killing parts which are sound. But if the patient be "horribly afraid" of sharp instruments, and more especially if the disease be superficial, caustic may be employed with advantage. In superficial suspicious ulcers of the prolabium, we have often used the potassa fusa with the best effects. 6. Nitrate of silver (used not as a caustic), leeching, and general treatment will often succeed in curing affections of the tongue, lip, and cheek, which simulate cancer, but which the experienced eye discovers not to be so. And we are inclined to suspect that M. Lisfranc might beneficially apply this principle to his own practice more extensively than he has hitherto been in the habit of doing. 7. "All is not gold that glitters." Desault and others who fancy they have cured cancers by compression are mistaken; they have, by the combined agency of ulceration and absorption, dissipated a morbid product, but it was not carcinomatous, though at first it may have seemed so. 8. Will cancer cure itself? No! A clap will run itself out; tic douloureux will disappear spontaneously, after the whole pharmacopœia has been swallowed fruitlessly; a scrofulous tumor will commit suicide; a stone in the bladder may find its own lithrotriteur; but no one, unaided, shall rid himself of cancer. 9. In obstinate ulcers simulating cancer, M. Lisfranc advises mercurial treatment, although no primary venereal affection have attacked the patient previously, having some vague notion about the hereditary qualities of this much-abused disease; as thus—the father probably had syphilis, he transmitted the virus to his fœtal son, the virus remained latent in the system of the latter for many years; at length it has ventured out; but, as if still ashamed to show itself, comes in disguise; natheless, the mask is to be plucked off, and the sly intruder is to be knocked on the head by means of its ancient and inveterate foe, mercury! This requires no comment. 10. Marked and extreme cachexy is not of itself sufficient to contra-indicate removal of cancer by operation. Granted; but when we are told that glandular enlargement in connexion with the original disease, combined with marked cachexy, is still no bar to operative interference, we protest most stoutly against such dangerous doctrine, and regret extremely that M. Lisfranc should be thus an encourager of rash and hopeless knife-work, while his brethren, at least on this side of the channel, seem to have come with one consent to a diametrically opposite practice, more consistent with humanity, good surgery, and common sense. By such a combination as we have just mentioned proof is plainly afforded that the disease has obtained a firm hold of not only the part and its neighbourhood, but of the whole system, that it is consequently impossible for any operation to cure the disease; and surely protraction of life and alleviation of suffering are more likely to be obtained when the malady is allowed its own way, than when its inroads on the frame are assisted by the shock of a severe operation, and by the ghastly draining wound which must inevitably result. Not only is the patient thus made doubly a sufferer, but by the failure which is inevitable, foul discredit is done to surgery. And this practice is advocated by M. Lisfranc ! *et tu Brute !* 12. Cancers of the skin, and those occurring in adventitious tissues are the least likely to return after operation. 13. Our author believes cancer to be in many cases hereditary, and yet, other circumstances being favorable, does not hold a well-grounded suspicion of such hereditary tendency to forbid operation. 14. A carcinoma stationary, and causing but little annoyance, in a patient of a far advanced age ought not to be interfered with. The disease has taken a nap, and it would be folly to disturb its slumber ! 15. The superficial tubercles often found clustering around a carcinoma are not in the substance of the skin, as generally supposed, but subcutaneous, with the superimposed integument stretched and much attenuated. 16. When cancer returns is it possible to destroy the formation without either knife or caustic? M. Lisfranc's answer is affirmative; but the proof brought forward to support the bare assertion is not satisfactory. 17. The swelling of carcimona does not consist *entirely* of carcinomatous tissue; the circumference is often composed of simple effusion and deposit, as in swellings of inflammatory origin. On this general fact our author founds the following neat surgical maxim. When a carcinomatous tumour is of such a size as to render extirpation dangerous, either by the mere extent of wound, or by encroaching upon important vessels, viscera, or cavities, neither rashly operate, notwithstanding, nor yet abandon the patient to his fate, provided there be no other circumstances unfavorable. But adopt such means as are likely to dissipate ordinary hypertrophy of natural tissue; occasional and moderate depletion in the neighbourhood will remove the vascular excitement, and diminish the heat and pain; discutients, the best of which class here is gentle pressure, will gradually remove the deposit; but it is obvious that the utmost caution is necessary in carrying out the latter indication, otherwise the malignant action will be stimulated, and it is our object to have nothing to do with that at present. By due persistence in such preliminary treatment, the tumour will be ultimately reduced to what is merely carcinomatous—probably one half, or nearly so, of the whole former bulk. And now the operation becomes not only practicable, but comparatively simple and safe. 18. However, let the superficial observer beware of imagining in such cases that because one portion of the swelling has yielded to simple means, the rest will follow its example; acting on such a supposition, as we have already seen, he will at a certain point cease to do good, and then go on inflicting positive harm; the *common* product may be dissipated, the *perverted* cannot, and the latter will be but stimulated to further development by the unsuccessful attempt. 19. M. Lisfranc agrees with other good surgeons in cutting wide of the tumour, leaving about half an inch of sound tissues all round; and also in looking carefully in the wound for little tubercles which are apt to be overlooked, and which, if left, are certain to regerminate the disease. 20. A carcimona taken away, and cicatrization completed, the surgeon's duty is not ended. Relapse is probable: certain means tend to prevent it, and these are to be sedulously employed. Chirurgery consists not solely of "carpenters' work," but both in science and practice goes hand in hand with medicine. The patient is to be reassured, and kept from brooding in despondency on anticipated relapse; every one knowing that mind and body react closely on each other. If congestion threatens, bleeding is prudent, depleting or revulsive, according to circumstances. Excretions and secretions are to be closely looked to, and put to rights if necessary. Our author has not forsworn the ancient allegiance to hemlock as supreme in this affection, and accordingly gives a grain of the powder in pill every morning; the dose is doubled at the end of the first week, tripled at the end of the second, quadrupled at the end of the third; and this last dose is continued. He believes this medicine to be "antinervous and discutient," and only in very few instances has found it irritate the digestive organs or excite the nervous system; in such cases of course its use is contraindicated. Against the glandular affections not avowedly carcinomatous, he directs the hydriodate of potash; this is doubtless good practice, but would become the opposite did malignancy declare itself in the tumours. Friction around the cicatrix, and compression of it are recommended, but we dread both; they are apt to overstimulate, and all stimulation here is dangerous. When he adds that "all causes capable of irritating the cicatrix are to be avoided," we think he contradicts himself, and therefore readily agree with him. Motion, especially, should for a long time be sedulously avoided; the difficulty of obtaining this indication complete, after removal of carcinoma or cancer of the lip, is the main cause of frequency of relapse after this operation, and a good illustration of the advantage of rest. 21. In hopeless cancers, starvation, it is well known, does harm. But after removal of cancer, and during hopes of permanency of cure, the diet cannot be too guarded, all exciting aliments being religiously avoided.

*On the dressing of wounds.* In this particular the French are behind us. In all wounds, it is the usual custom in Paris, to plaster on what they term their "first dressing," and leave it undisturbed until it is loosened by a soaking suppuration. M. Lisfranc naturally objects to this concealment of the wound during an important and often critical period, and wishes to see what it is doing, after the first twenty-four hours at latest. To pull off dry lint, however, at that time, would inflict great pain on the patient, and do a positive injury to the part. He accordingly makes *his* first dressing of easy removal, by larding well its surface, and taking care that it is sufficiently large to rest with its margins on sound skin, apertures being cut in its centre for the escape of fluids from the wound. This he considers a great improvement. We would be sorry to have it in exchange, however, for the simpler and more agreeable, as well as more efficient treatment, commonly known as the "water dressing," cold or hot, simple or medicated, now in use by the better-informed surgeons in this country, and for which they are indebted to Liston, Macartney, and others—zealous and successful improvers of this important department of practical surgery. We cannot help thinking that climate and season are not alone to blame in the marked failure of union by the first intention in the French capital; with their present system of dressing, it would probably be surprising did suppuration ever fail to be established. And as M. Roux occasionally favours us with a flying visit, we would suggest that he should draw an impartial "parallel" on this point; as we presume to think it might not be without advantage to Parisian surgery. Nay, we are free to admit, that even in this country the improved mode of dressing is not yet sufficiently attended to. And accordingly we have felt it to be our duty to place before our readers the following condensed view of the practice, partly for the benefit of the future parallelist, partly for the sake of our junior professional brethren. (*a*.) In regard to adhesion of incised wounds, delay in approximation is advisable. Cold-water dressing is applied until bleeding has ceased. Then the wound may be closed; but it is better to wait a little longer until the reparative process has commenced. This preferable condition is indicated by a glazed appearance of the cut surface. (*b*.) Delay, and the cold applications are, besides, useful in preventing secondary hemorrhage. Should this occur, the open state of the wound is favorable to the adoption of means necessary for its arrestment. (*c*.) In effecting approximation, stitches may be employed when necessary; but they should be few, and in all cases their use is temporary. In a great number of instances they are entirely dispensed with. The principal and permanent retentive means are slips of non-irritating isinglass plaster.* And as soon as these have been applied, and become fixed in their hold, all sutures are removed. The harelip operation, and some other wounds, are exceptions. (*d*.) The isinglass plaster, being translucent, admits of a constant and complete surveillance of the uniting process in every part of the wound. It does not irritate the surface on which it is applied, is very adhesive, and seldom, if ever, requires renewal during the cure. (*e*.) No other dressing is applied. When coaptation has been effected and made permanent, all the manipulation necessary to adhesion is accomplished; dressings additional to the plasters, therefore, can do no good, may do harm, and are to be avoided. Cleanliness of the part, by gentle and occasional wiping, not of the wound but of its neighbourhood, is all that is further requisite. (*f*.) By this mode of dressing, the occurrence of adhesion is rendered much more probable; the patient is saved much pain and irritation, and the surgeon is freed from infinite trouble and annoyance. Should adhesion fail, the parts are in a much more favorable state for assuming the other process of union than they would have been, in similar circumstances, under the old system. (*g*.) In regard to union by the second intention, no stitches are employed. Approximation does not require to be complete, and the cure is effected by simple replacement and attention to position. (*h*.) If acute inflammatory action threaten to prove excessive, active and timely antiphlogistics must be resorted to, according to general principles. (*i*.) Usually, water-dressing is the only application, unless during the latter part of the cure; at first continuously cold, to retard and limit the inflammation; then hot, so as to moderate the excited action, at the same time favouring the escape of foreign bodies, if there be any; afterwards it is tepid and comfortable, as simply detergent. When gentle stimulation of the granulating surface is required, towards the end of the cure, the water is medicated by stimulants, as the sulphate of zinc, proportioned in strength to the exigencies of the case. (*j*.) Heavy, fetid, cumbrous poultices, and greasy, rancid, irritating ointments are superseded. (*k*.) In the last stage of union, both by the first and by the second intention, support, with mild and uniform pressure, is not unfrequently advisable. It is effected by plasters, by bandage, or by both. (*l*.) The process of union by the second intention may sometimes be dexterously supplanted by that of adhesion. The

* Liston's Plaster, vide our last Volume, p. 265.

period when this can be accomplished is, when the vascular action has subsided from the inflammatory and suppurative to what is simply essential to reproduction. When the young and active granulations are then brought into close contact, they quickly coalesce, a great part of the uniting process having been effected previous to apposition, and the secretion of pus having very much diminished. (*m.*) Two prominent exceptions to the preceding rules are,—1. When the cut surface is such that every point can be placed in close and accurate contact, without the risk of coagulum or any other obstacle to adhesion being interposed; then, twisted sutures constitute the whole dressing. 2. When the wound is so situated that neither plasters nor twisted sutures can be applied, then the common interrupted sutures must be employed, as few in number and of as short duration as possible.

*On the treatment of Sprain.* There is no difficulty in obtaining plenty of remedies in this case. The only nicety and skill is in knowing the proper time at which to use them. Cold, leeches, pressure are excellent means of treating sprain, but only when each is in its own place. The more important indications of treatment are simple; to prevent, diminish, or remove inflammatory action; and to favour reabsorption of the effused fluids. For the first indication leeches are useful, but they are totally inoperative when applied immediately after the injury, as is often the case, with the view of abstracting the actually effused blood; moreover, their bites may lead to unhealthy suppuration, and even to gangrene; for it is well known how imprudent it is to expose extravasated blood to atmospheric accident. M. Lisfranc supersedes leeches by venesection, this being less liable to injure locally, being equally effectual in combating inflammation, and being likely to contribute somewhat to the fulfilment of the second indication; because, according to the theory of M. Magendie, when we act directly upon the venous system, and suddenly diminish its amount of blood, the veins become "thirsty of liquid," especially if the patient himself drink sparingly; and then they are supposed not to be unwilling to slake their thirst even on the stale fluids of effusion; hence absorption is likely, under such circumstances, to be speedy. This, for removal or subjugation of excited action. To prevent it, cold is valuable; and just for this reason the proper time to employ this much-abused remedy is immediately after the accident, before inflammatory action has been lighted up; after this has commenced to form, persistence in the use of cold is downright folly; it must be superseded by poultice, fomentation, and other means suitable to combat a growing inflammation. After five or six days the swelling is diminished, and has become more œdematous than inflammatory, the effusion is partly reabsorbed, and the pain is much abated. In short the excited action has passed away, and now is the time to abandon depletion and fomentation, and have recourse to friction and bandaging; in order to expedite fulfilment of the second indication—restoration of the parts to their normal condition. But to use either friction or bandaging at an earlier period, as is very commonly done, is plainly to thwart nature and aggravate inflammation. Above all things let *rest* of the injured part remain complete and undisturbed throughout the whole period of excitement, otherwise that period will prove unnecessarily protracted; and this is a point, by the bye, on which our author is culpably silent. Neither let the patient have an imprudent haste to be well, but let an almost imperceptible gradation bring back the part to its former functions. By following such a mode of treatment in sprain, M. Lisfranc asserts, and we believe him, that he has never failed to effect a cure, within a comparatively short period, and to prevent weakness of the joint which might otherwise leave it prone to repetition of the injury. In fact he declares himself happily ignorant of this " pretended feebleness" which others bemoan so much. In some cases the sanguineous or sero-sanguineous effusions resist the ordinary routine of treatment. Against such obstinacy M. Lisfranc directs purgatives and diuretics, according to the theory we formerly noticed; to the latter we have not the same objection here as in the case of fracture.

*Burns.* 1. M. Lisfranc adopts nearly the same classification of this class of injuries as did Dupuytren: *a.* Mere erythema, resulting from slight injury of the mere surface. *b.* The rete vasculosum is fairly involved, and the inflammatory symptoms are more decided and serious. *c.* The chorion has been reached, and is more or less injured. *d.* The whole thickness of the skin is killed outright. *e.* The subjacent tissues are involved, and more or less charred. These five forms of the injury may be found all united in one instance, the fifth usually occupying a central position. 2. At four periods the patient's life is in danger; during the period of irritation or shock, the first effect of the injury; of inflammation and its direct accompaniments; of suppuration; and of prostration or collapse. 3. Our author justly reprobates the empiricism, too common in our profession, of imagining certain applications to be specific in all cases of injury by fire, and using them accordingly; and insists that the treatment, both local and general, shall be varied according to the nature of the injury, and the progress of the case. Cold, for example, can only be of use as a preventive of inflammation, or rather as a means of limiting its intensity; the time for its application is immediately after the accident, during the period of incubation; after a few hours have elapsed inflammation has begun and is advancing, and then its farther use not only ceases to be advantageous but becomes absolutely hurtful. It must give place to the suitable antiphlogistics. In irritable subjects too, especially if the internal organs seem prone to the assumption of morbid action, continued application of cold to an extensive burn, even of the first class, must often be omitted, or at all events managed with the utmost caution, otherwise the most serious results may ensue. Cotton and flour are valuable applications to burns of the first and second classes; but to them they should be limited. For if employed in those of a severe nature, they not only injuriously conceal parts where important actions are in progress, requiring supervision, promotion, or repression —but also, by retaining discharge, become filthy, disgusting, and perhaps the source of a fatal irritation to the very textures which they are vainly destined to protect. Bloodletting is often our most trusty means of meeting the brunt of a violent inflammatory attack, as is far from unfrequent in those of the third class; but after suppuration has been fairly established, a prudent man will draw no more blood, knowing that the period of its usefulness has gone by, and that now the system will have to muster all its resources, in order to bear up under an exhausting discharge. Stimulants and tonics are most useful to assist nature under such circumstances, and the former are often most valuable in warding off an earlier danger from the first shock of the injury; but after reaction is over, and inflammation begun, what but harm can result from the use of either? Local stimuli are of the most signal service in obviating the sluggish tendencies of the sore that results from the separation of the charred sloughs; but how they should be otherwise than detrimental previously to this separation we cannot imagine. Sedatives and narcotics are of good service in the period of excitement; in that of collapse they are more likely to prove fatal. Such principles seem consistent at once with good surgery and with common sense, yet how often do we see them transgressed in the every-day practice of those who in other matters evince a far wiser experience. Laziness we fear is inherent to humanity; and we are all too apt to get into a routine system of treating diseases by their names, and not by the varying symptoms and indications as they appear; a fault not more common than absurd. 4. Meddlesome surgery is our abomination; and accordingly we are glad to find M. Lisfranc strenuously forbidding all attempts to pull away the yet-fixed eschars; let the detached portions be separated as soon as possible by the forceps and scissors; but leave the process of detachment to the more competent hands of nature. 5. Sometimes pus accumulates under the eschars, and according to the general rules of sound surgery is to be evacuated by suitable incision, at an early period; otherwise serious injury will be done to the parts beneath. But reckless scoring of the sloughs as practised by some, with the hope of giving greater efficacy to their stupid "antiseptics," is not only useless but dangerous; for important nerves, veins, arteries, may thereby be wounded, which might otherwise have escaped all but intact from the effects of the injury, of the inflammation, and of the subsequent sloughing. Nature has wisely endowed the arterial tissue especially with the remarkable power of resisting disease, but she is unable to contend with disease and with the relentless steel of a reckless knivesman. 6. The principal object of our author in this chapter seems to be to introduce to our notice *his* panacea for burns—the chloride of soda. Of the chloride of lime he has also a high opinion; but of the two, prefers the former. An abridgment of its character is as follows. It is astringent and sedative. It not only delays the approach of inflammation, but even dissipates it almost entirely. By mitigating the impression on the nerves, not only is pain assuaged but constitutional symptoms materially modified, if not wholly prevented. When there is no slough, breach of continuity caused by the burn is quickly covered by a plastic exudation, becoming rapidly organized, and resembling false membrane; and almost the whole credit of this production is attributable to the chloride. After sloughs have separated, and granulation is considerably advanced, the chloride is again able to finish the process with eclat by the effusion and organization of this membranous product; a process similar, apparently, to what in this country is termed the modelling. The cicatrix thus obtained is more solid than that resulting from the ordinary means; both in this new matter, and in the original tissues that surround it, there is much less tendency to contraction, otherwise so fertile in deformity; indeed so self-sufficient is the creation by the chloride, that the original tissues are scarcely asked to contribute anything by accommodation towards repair of the breach. It is at the same time admitted that the chloride used during the supremacy of a powerful inflammation is decidedly injurious; that employed with the view of arresting gangrene so caused it is at least ineffective; and that it is most prominently useful in injuries of the first and second degrees. With such exceptions, however, it is *the* remedy in all burns; subduing all untoward symptoms, local and general; shortening the period of cure; expediting separation of the sloughs; producing more favorable cicatrices; and often preserving life, which would doubtless have been lost under the ordinary mode of treatment. This is no lukewarm praise: may experience confirm its truth! 7. The chloride is used in solution; the ordinary proportion is one part of the concentrated solution to thirteen of water; but this is to be varied according to the effects produced. The test of its efficacy is that its application produces an itching and slightly painful heat in the skin, of not more than a quarter of an hour's duration; more or less than this is either hurtful or inert. 8. So salutary are the effects of the chloride in the eyes of our author, that he enjoins even removal of cuticle in order that the application may prove more direct and consequently more efficacious. On behalf of the cuticle we protest against this principle, believing nature's to be at least as efficient a protection to the tender surface as any of artificial construction; and that therefore it ought not on light grounds to be superseded. 9. The mode of application is as follows: a compress covered with "cerat de Galien," and perforated at several points, is placed over the whole surface of the burn. Above it is laid a mass of charpie, at least two inches in thickness; this is saturated with the solution of the chloride and covered with dry compresses; the whole is fixed by a bandage. The apparatus is sprinkled with the solution every two or three hours; and every twenty-four hours the dressing is renewed. 10. After burns of the fourth and fifth degrees, the cicatrices are apt to prove vicious, with more or less deformity. In the treatment, the probability of this must always be kept in view, and endeavours made to produce a large cicatrix composed chiefly of newly created matter. Attention to position will sometimes suffice to fulfil this indication; in other cases, pressure, traction, and escharotics have to be called into use; and in not a few all efforts and skill will prove unavailing. One fact should never be forgotten; the tendency to contract does not cease with the completion of the cicatrix, but continues for many months afterwards; and unless the preventive treatment, by position and otherwise, be continued patiently until the contraction shall have entirely ceased, deformity is certain to occur. In burns of the face, contraction of the original integument is combated by centrifugal traction effected by strips of adhesive plaster, and sometimes with considerable success. M. Lisfranc proposes a modification of this means, likely to prove more effective. Around the breach hairlip pins are applied, their blunt heads projecting over the sore; a plaster, split to near its end, is hooked on each of these, laid down, and held in its place by a general retaining strip laid in an opposite direction. These needles and plasters, proportioned in number to the size of the sore, keep up a traction from the centre to the circumference, antagonist to the natural tendency of the ulcer. Such treatment is of course incompatible with rapid healing; but of the two evils we are undoubtedly to choose the least. Redness of the cicatrix is successfully combated by steady and uniform pressure on the part; and a similar means proves very effectual in reducing irregularities of the cicatrized surface to one equal level, whether the result of burn or of smallpox: in the face a kind of mask is the most suitable agent of pressure; and it is the more likely to succeed the earlier the time of its use. 11. Position, traction, pressure, not unfrequently fail to prevent hard, prominent, contracting cicatrices. Excision is the remedy still within our reach. On this subject, however, nothing new is brought forward by our author; nor is his hope of success more buoyant than that of his less mercurial brethren; there is no reason therefore why we should enter farther on a subject so unpleasant and unsatisfactory. In the face he agrees that no such operation should ever be attempted.

*On venesection at the bend of the arm.* 1. This simple and sometimes fatally-facile operation, though conducted on much better principles than formerly, is yet not unfrequently followed by local accidents. 2. When detection of the veins is difficult, the ligature should be applied for half an hour or even an hour, the patient meanwhile frequently contracting the muscles of the forearm. 3. Often there exists an imperious necessity for general bleeding, while at the same time it is impossible to see or feel veins in the leg, foot, wrist, forearm, arm, or neck. Instead of being constrained to perform arteriotomy, which may seem an insufficient substitute for phlebotomy under the circumstances, it is better to cut down on the cephalic vein, as it passes between the deltoid and large pectoral muscles; the operation is attended with no difficulty or danger. 5. Meddlesome fingering of a wounded vein, with the view of compelling it to give forth blood, is forbidden both by reason and experience; phlebitis, always dangerous, is almost sure to result; it is consequently far better to make a fresh opening at another point.

*On deligation of arteries.* 1. In this chapter, M. Lisfranc commences badly. He has too many instruments. "Straight bistouries both sharp and blunt pointed, dissecting forceps, blunt-pointed scissors, straight and crooked, flexible "sonde cannelées," needles, and several ligatures of different thickness and breadth," do not constitute the most promising array of implements for attacking large arterial trunks, at least to the English eye. With a scalpel, forceps, one needle, and one ligature neither thick nor broad, all the requisite manipulations can be readily and safely performed. And we hold it to be one of the soundest maxims of surgery, that instruments, if efficient, cannot be too simple and few. 2. National prejudice leads M. Lisfranc to omit all mention of John Hunter in the improved operation for aneurism; he wishes to divide the honour of that discovery between Ambrose Paré and Anel, both excellent men in their way, but neither entitled to dispute Hunter's claim to the operation which in this country uniformly bears his name; unless indeed, according to the principle inculcated in a common domestic proverb, concerning the predilection entertained by certain aquatic fowls towards the assimilating organs of fishes; but which being somewhat coarse in the vernacular, need not be more plainly exhibited in these pages. 3. In operating with the view of placing a ligature on an arterial trunk, M. Lisfranc practises a preliminary movement, not thought necessary in this country, except in the case of puncture of the artery aforesaid, viz. pressure by an assistant at a distance from the scene of operation. He must be a very rash or unsteady operator who wounds, so as to cause hemorrhage, the artery which he exposes for the purpose of deligation; and moreover, by pressure, such as arrests the arterial flow, pulsation of the vessel is lost, which might otherwise have proved a very important guide in the earlier part of the incisions. 4. For compression, M. Lisfranc agrees with the majority of modern surgeons in preferring the fingers of a steady and good assistant; such pressure is more exact, more limited, less severe, and more easily shifted if necessary, than that of any tourniquet; inflicting less injury to the part compressed, and, in the case of large wounds, causing less injury to the system by sparing the loss of blood. In not a few situations, also, pressure can be thus applied, efficiently, when all tourniquets are plainly inapplicable. 5. Regarding the external incision, in the operation for aneurism, M. Lisfranc wishes to carry out the principle of reducing everything to an exact scale of inches and lines, a mechanical ingraftment upon operative surgery, of which he seems particularly fond; in fact, according to him, a side-pocket containing a foot rule of measure ought to be as indispensable to a surgeon as to a carpenter. Now, we are here opposed to this mincing exactitude, and are ready to assert that the superficial incision can scarcely be too free. We have repeatedly witnessed very palpable proofs of the inconvenience and actual danger of too-limited an incision; the operator is cramped for room, has a difficulty in reaching the vessel; having come to its whereabouts, he runs a risk of unnecessarily detaching it from its connexions in order to make certain of its identity, and accordingly performs the operation not only clumsily and tardily, but with a good prospect of secondary hemorrhage all in due time. To avoid such embarrassment, the superficial incision, extending through the skin and subjacent cellular tissue, ought not to be measured by lines or units of lines, but by the eye and experience of the operator; who, if he errs at all at this point, should lean towards the side of unnecessary amplitude. As he advances in his dissection, the limits of the wound should gradually contract; but as they do so, he will often have occasion to congratulate himself on the liberality of his first use of the knife. It makes little difference in the progress of the subsequent treatment; on the contrary, we are inclined to think a free incised wound, such as we recommend, more likely to unite by adhesion, than one which in its form resembles the "punctured," and which by dint of groping, pressure, and laceration of its tract, can also lay some reasonable claim to the designation of "contused and lacerated." 6. In speaking of venous hemorrhage, resulting from accidental wounds of the concomitant veins, during the dissection, M. Lisfranc recommends pressure as the hemostatic; and, failing that, then ligature of the vein at the bleeding point. Now, we can hardly conceive the possibility of an unavoidable necessity for deligation of a vein, and that is all; and we almost shiver when M. Lisfranc talks so coolly of the alternative. The least result from such a procedure is the occurrence of the mildest and most favorable form of inflammation in the venous coats so deligated, occasioning permanent obstruction of the canal. This taking place simultaneously with occlusion of the arterial trunk, so interferes with the circulating functions, as to render invincible gangrene of the whole limb fearfully probable. But a higher grade of phlebitis is more likely to be excited, inducing death, not of a part, but of the whole, and that with appalling rapidity: and yet a surgeon is found to contemplate such contingencies with apparent unconcern. This is surely a dangerous example in clinical surgery; for, in our opinion, no more important precept can be impressed upon the pupil than high veneration for the venous texture, avoiding interference with it in all his operations as much as possible, and never dreaming of a ligature unless in the extremity where pressure fails, and life must be saved from hemorrhage at all risks, and that will be but seldom. 7. Should the artery prove yellow in colour, thereby intimating an unsound condition of its coats, we are recommended not to open the sheath, but to include this as well as the artery in the noose of the ligature. Against this precept also we protest, believing that be the arterial coats sound or unsound, the most likely mode of obtaining their salutary adhesion is to include them alone, taking especial care at the same time that the passage of the ligature for this purpose shall disturb the cellular connexions as little as possible. 8. In opening the arterial sheath, M. Lisfranc prefers his own finger nails to all other implements. Again we disagree with him; and prefer the scalpel with which we conduct the whole of the dissection, aided by the dissecting forceps. The scalpel cuts, the nails tear; an incised wound is capable of healing by adhesion, a lacerated one cannot, and must suppurate; adhesion is especially desirable in wounds implicating large arteries; suppuration, extensive or cooped up, is imminently dangerous; every means, therefore, should be adopted likely to conduce towards union by the first intention, and tearing is certainly not one of them. 9. The artery having been exposed, a "sonde cannelée" is passed beneath it, and along the groove of this an armed needle is subsequently introduced. This is not only an unnecessary complexity of instruments for the attainment of one simple end, but likewise occasions a detachment of the arterial coats from their cellular connexions to such an extent, as is most likely to prove fatal to adhesion and obliteration, and very favorable to ulceration and hemorrhage. A few cautious touches with the knife's point, on each side of the part destined for deligation, followed by the gentle insinuation of an ordinary aneurism needle, neither too sharp nor too blunt in the point, is surely both more simple and safe. 10. Previous to deligation, should "small nervous filaments or little veins" be found included in the noose, they are to be tied without remorse, according to M. Lisfranc, and no attempt made to disengage them; otherwise we might injure the cellular coat of the artery, thereby encountering the risk of "grave inconveniences." Once more we dissent. It is quite possible to disengage them; in fact to tie the artery and the artery alone, neatly and cleanly, without otherwise injuring any of the coats of that artery. There may be difficulty, when using one or more "sonde cannelées;" there is none, inseparable at all events, with the ordinary aneurism needle. In any case, the "grave inconveniences" of an attempt are not likely to equal those of the inclusion of nerves and veins. 11. Before completing the deligation, before tying the knot, M. Lisfranc toys with the vessel for some time, repeatedly elevating it by the ligature, yet loose, and applying pressure with the fingers, to ascertain the effects on the aneurismal tumours. Much, if not all, of this is unnecessary. An anatomist and surgeon scarcely requires this test to assure him he has obtained the proper vessel; or at all events, but little of it will suffice. So much as is required by M. Lisfranc seems likely to interfere injuriously with the important arterial connexions, thereby favoring suppuration and ulceration, if not actual sloughing. And we may here mention that in each of the few cases of ligature of important arteries by M. Lisfranc, detailed in this chapter, secondary hemorrhage occurred. 12. M. Lisfranc uses ligatures which are at first flat, but become round when knotted; they always divide the internal and middle coats of the artery. The advantages derived from their being broad at first is not mentioned. He also seems to place some faith in the superiority of animal ligatures, believing in the possibility of their disappearing entirely by absorption after a long period. We are far from possessing so lively a faith on this point. We have seen the noose of a catgut ligature emerge from a wound in which it had been confined for months, exactly the same to all appearance as when it first entered. Because a wound heals, and ligatures are seen no more, that is no sign that they have been absorbed; they may either have escaped furtively and unnoticed, or they have come to an amicable arrangement with the neighbouring tissues, by them have been kindly provided with an investing cyst of their own, aud have comfortably established themselves accordingly in a permanent residence, as do bullets and other foreign bodies less adapted for such mutual accommodation. 13. But we cannot help suspecting M. Lisfranc, after all, of an involuntary and lurking doubt of the alleged good qualities of animal ligatures; for by and by, we find the following: "I think, along with many practitioners, that the ordinary ligature, one of whose ends is cut off close to the vessel, is preferable to all others." Doubtless. And glad we are to find him discountenancing the absurd practice of leaving a deserted noose, with both its ends cut away, thrown entirely upon its own resources for restoration to the external world, from which it is thus effectually excluded, at least for the time. The only chance of immunity from mischief in such circumstances, is encystment of the foreign body, as already mentioned; but in the great majority of cases, nature sooner or later ejects it by the only means in her power, suppuration; it is, in short, compelled to work its passage out. And often have we seen stumps reported cured, soon come back to treatment with deep-seated abscess, creating no little disturbance both general and local, and not to be quieted until after expulsion of the sole cause, the unabsorbed and undissolved noose. The leaving of one end pendent will not interfere seriously, if at all, with union of the wound; and when that is obtained, it may, under such circumstances, be happily regarded as permanent and secure; moreover, secondary hemorrhage need not be apprehended from deep-seated secondary abscess. 14. The application of two ligatures, with intermediate section of the vessel, as advised by Mr. Abernethy, is recommended by M. Lisfranc only in certain situations, as, for example, when the superficial femoral is deligated. All the advantage likely to result from such procedure appears to us to be obtained by suitable flexion of the limb; and hence we hold that an unnecessary extent of injury is inflicted on the vessel by such mode of operation. Under certain circumstances its use becomes inevitable; when from some cause or other the surgeon has detached the exposed vessel more extensively than is required for the mere passage of the aneurism needle, then by all means let him not neglect to apply a ligature at each extremity of the detached portion; and he may then practise intermediate section of the vessel or not, just as his fancy may dictate. But in all other cases, the single ligature, well applied, is infinitely preferable. 15. M. Lisfranc fears that sometimes the ligature perforates the artery prematurely, before consolidation of the canal has been completed, consequently entailing hemorrhage; but it requires further proof to fasten the blame of such an accident solely on the ligature; the arterial coats may be at fault, and in the majority of instances of such hemorrhage we are inclined to believe they are so. Consequently we hold M. Lisfranc's censure of the ligature on this score to be not proven, and probably unjust. He, however, countenances none of the "serre-nœuds," "ligatures d'attente," and other clumsy means intended to obviate such disaster, and which only realize the old proverb, in most other cases happily almost obsolete, that "the cure is worse than the disease." Temporary ligatures, too, he holds in no estimation; "sound surgery commands that we leave the ligature in its place, until its spontaneous separation." 16. When the arterial coats are obviously diseased, M. Lisfranc prefers compression to ligature, and talks vaguely of plugging the vessel, should hemorrhage threaten or occur. We, on the contrary, think such pressure and manipulation well calculated to ensure the very accident he wishes to avoid, and believe that a well-applied ligature affords the only chance of a successful result, even in such cases. 17. M. Lisfranc well insists on the propriety of avoiding all moral and physical causes likely to accelerate the circulation suddenly and much, at the time when the ligature is about to separate; otherwise the obliterating process may be broken up and hemorrhage ensue. 18. In 180 cases of the Hunterian operation for aneurism, collated by our author, hemorrhage took place in 32; that is, 1 to 6. In the majority, the bleeding occurred between the 16th and 24th days; the earliest hemorrhage appeared at the end of 24 hours, the latest at the end of the second month. The number of deaths was 43, or 1 to 4; other causes than hemorrhage having been at work towards the fatal issue. 19. A case of femoral aneurism is detailed, in which M. Lisfranc tied the external iliac. Two excellent precautions are given, observance of both of which is incumbent on every surgeon: (*a.*) Never to perform deligation of any large artery on account of aneurism, until the patient has been some days in hospital; in order to ascertain whether such residence agrees with him or not. And we would add, in order to afford an opportunity for preliminary treatment, tending to improve the general health, to tranquillize the circulation, and to avert the accidents incident to the operation. (*b.*) The second precaution we quote verbatim. "Notwithstanding my self-confidence, obtained by practice and experience, I have not thought myself warranted in performing so serious an operation without examining anew what I have so often seen and demonstrated. Thus, you will bring clearer and more accurate ideas into the practice of surgery, not imitating the crowd of imprudent operators who put their hand to the work without being sufficiently aware of what they ought to do. Never act thus when you have serious operations to perform, the malexecution of which may cost the lives of those who have confided in you." Such sentiments coming from a French surgeon are delightful, for they lead us to hope that the time has gone by when human life was by them held of but little account. 20. In exposing the external iliac, M. Lisfranc adopts a line of incision intermediate between those of Abernethy

4291

and Cooper. The outer extremity is situated two lines above and an inch within the anterior superior spinous process of the ilium; the inner is placed an inch and a third without the spine of the pubes, and about an inch above its level. Thus he avoids the perpendicular section of the fibres of the abdominal muscles, which Abernethy's mode entails, and which by weakening the abdominal parieties disposes to hernia. His incision is much less extensive than that of Cooper, and consequently is not so likely to injure the spermatic cord, or the epigastric and anterior iliac arteries. The preferable point of deligation is about midway between the origins of the internal iliac and epigastric arteries, thus escaping the disadvantage of placing the ligature immediately beneath a collateral branch. 21. Of the mode in which he proceeds to dress the wound we cannot approve, for the reason formerly stated; first a compress well larded, then charpie, and lastly a poultice; all at once! and this for a wound in which it is of much importance to obtain union by the first intention. No wonder that in the subsequent history of the case we hear a copious suppuration spoken of as having threatened to "run away with" almost the whole of the external oblique muscle. 22. To another procedure we also demur. Immediately after the operation "the limb is enveloped in heated linen cloths, which are renewed from time to time." What more likely cause of gangrene is there, after ligature of the principal vessel, than stimulation of the limb by heat or friction? By the operation the vital powers of the whole parts beneath the point of deligation are materially diminished, and they are not recovered till after a considerable time; and yet here is a surgeon of much experience directing us to stimulate such parts, whereby we do our utmost to bring on an excited action which they have not the power to control, and before which they must consequently fall an almost unresisting prey! "Une potion antispasmodique, de l'eau de gomme, de la limonade edulcorée," will scarcely compensate for so flagrant a breach of sound surgery; the patient happily escaped, however, from the risk he was forced to encounter, gangrene did not take place. 23. On the 46th day after the operation symptoms occurred, alarming in appearance though not in reality; deception against which it is well to be on our guard. The patient, in a state of obvious anxiety, reported that for several hours he had felt strong pulsations in the wound. On exposing it, the abdominal parietes at that point were seen gently heaving; four or five undulations occurred in succession, ceased for an instant, and were then repeated. At first M. Lisfranc felt much alarmed; but the intermittence of these movements, and their want of synchronism with the heart's action, showed they were not arterial; they proved to be muscular, and soon disappeared. 24. M. Lisfranc justly exults in the happy combination which has taken place between medicine and surgery, and by which the latter is enabled to obtain a successful issue in cases far from promising, as was the one now in question; the patient "had pulmonary and cerebral congestions, hæmoptysis, pain, and tympanitis of the abdomen, and yet all these yielded to the medical treatment." But one of the auxiliaries to the art he does not mention, though not the least valuable in ligature of the large arteries; viz. the use of bleeding and other antiphlogistics, prophylactic and actual, both before and after the operation, more especially in the case of the common carotid. On this point the profession is much indebted to his fellow-countryman, M. Robert; and the subject has been further illustrated by Mr. Miller in the January number of the London and Edinburgh Monthly Journal of Medical Science. 25. A case is related in which ligature of the common carotid was performed on account of a tumour in the side of the face, and which proved fatal by hemorrhage from the wound. With the designation of this tumour we are somewhat wroth. It is termed a fungus hæmatodes. Now, the usual acceptation of that term implies, at least with us, a fungus, a fungoid protrusion, of a bloody appearance, and prone to hemorrhage; further, this is usually found associated with malignant formation, sometimes carcinomatous, sometimes melanoid, but most frequently encephaloid. To speak of a fungus hæmotodes, then, necessarily conveys the impression that it is not only prominent, bloodlike, hemorrhagic, but also connected with malignant disease. How such a tumour is to be benefited by ligature of the common carotid is a problem of rather difficult solution; and hence, at the outset of the case, we are led to suspect M. Lisfranc of villanous surgery. But the sequel proves favorable to his surgical reputation, though far from laudatory of his proficiency in pathological nomenclature. Here is his own description of the tumour: "It is composed partly of aneurismal vessels, partly of erectile tissue; it has no distinct capsule, and is merely surrounded by the ordinary cellular tissue condensed, from which fibrous prolongations traverse the interior." So it turns out to be an aneurism by anastomosis; and he has been but imitating the more successful practice of Messrs. Travers, Dalrymple, Busk, &c. in similar cases, and has not been tying the carotid for a malignant growth, as he has led us to believe, and as he tells us M. Berard has cruelly asserted. But he has himself to blame for having induced us to suspect his judgment, however temporarily; and ought not to abuse M. Berard, or any one else, for an error of his own making. 26. M. Lisfranc considers that false aneurism is not always formed immediately after wound of the artery. The artery does not necessarily bleed immediately after the infliction of a puncture; for this, covered by a neighbouring muscle, or filled by a clot, may be obstructed; a shifting of the muscular fibre, or a displacement of the coagulum, however, will subsequently bring on the extravasation of blood. The coats of an artery may not have been cut, but severely bruised; they inflame and slough; and on the separation of the slough, blood may escape from the pervious vessel, so as to constitute a false aneurism. Hence it is wrong in surgical authorities to make the broad assertion, unqualified, that false aneurism is the immediate result of injury; and that when swelling takes place some time after its infliction, this is invariably attributable to inflammation. The great rapidity of its formation, along with the absence of the usual signs and characters of inflammatory effusion, declare it to be aneurismal at whatever period it may occur. 27. With our author's pathology of false aneurism we fully concur, but object to the treament of an imaginary case of gunshot injury which follows. A ball has made an ugly and extensive wound in the inside of the arm; the arteries below the wound are pulsating normally; hemorrhage takes place and the ordinary hemostatics fail to arrest it; are we to tie the brachial artery above the wound? M. Lisfranc says No; and prefers energetic stuffing of the wound. We would say Yes; and having tied the vessel as we best could, would apply a *moderate* degree of pressure to the wound, placing unbounded confidence in a successful issue. Whereas, with the use of such severe pressure as our author recommends, we should be afraid of ulceration being induced in and around the injured vessel; consequently, on the loosening or ablation of the compress, we should be nervously apprehensive of hemorrhage; and it is most likely that our fears would be fully realized.

We have now reached the middle of the volume, and, with what we hope may be considered becoming discretion towards our readers, pause awhile from our labours. The celebrity of the author has perhaps led us into a more minute examination than is our wont. We have extracted not only what is original, but also what seemed likely to prove both interesting and useful to the majority of our surgical readers; and from that regard to the latter which our position naturally engenders, we have not permitted either our veneration for a justly great name, or the possibility of an imputation of presumption against ourselves, to deter us from freely animadverting on what we humbly conceive deserving of censure. In the same spirit we propose to continue our dissection of the remaining portion of the volume in our next Number.

---

